# The rare enterovirus c99 and echovirus 29 strains in Brazil: potential risks associated to silent circulation

**DOI:** 10.1590/0074-02760190160

**Published:** 2019-08-12

**Authors:** Adriana Luchs, Elcio Leal, Kaelan Tardy, Flavio Augusto de Pádua Milagres, Shirley Vasconcelos Komninakis, Rafael Brustulin, Maria da Aparecida Rodrigues Teles, Márcia Cristina Alves Brito Sayão Lobato, Rogério Togisaki das Chagas, Maria de Fátima Neves dos Santos Abrão, Cassia Vitória de Deus Alves Soares, Xutao Deng, Eric Delwart, Ester Cerdeira Sabino, Antonio Charlys da Costa

**Affiliations:** 1 Instituto Adolfo Lutz, Centro de Virologia, Núcleo de Doenças Entéricas, São Paulo, SP, Brasil; 2 Universidade Federal do Pará, Instituto de Ciências Biológicas, Belém, PA, Brasil; 3 Universidade de São Paulo, Instituto de Medicina Tropical, São Paulo, SP, Brasil; 4 Universidade de São Paulo, Faculdade de Medicina, LIM/46, São Paulo, SP, Brasil; 5 Universidade Federal de Tocantins, Palmas, TO, Brasil; 6 Laboratório de Saúde Pública do Estado de Tocantins, Palmas, TO, Brasil; 7 Secretaria de Saúde de Tocantins, Palmas, TO, Brasil; 8 Faculdade de Medicina do ABC, Programa de Pós-Graduação em Ciências da Saúde, Santo André, SP, Brasil; 9 Universidade Federal de São Paulo, Laboratório de Retrovirologia, São Paulo, SP, Brasil; 10 Blood Systems Research Institute, San Francisco, USA; 11 University of California San Francisco, Department Laboratory Medicine, San Francisco, CA, USA

**Keywords:** Enterovirus, echovirus, gastroenteritis, serotypes, deep sequencing

## Abstract

Human enteroviruses (EVs) are associated with a wide spectrum of human diseases. Here we report the complete genome sequences of one EV-C99 strain and one E29 strain obtained from children suffering from acute gastroenteritis, without symptoms of enteroviral syndromes. This is the first report of EV-C99 in South America, and the second E29 genome described worldwide. Continuous surveillance on EVs is vital to provide further understanding of the circulation of new or rare EV serotypes in the country. The present study also highlights the capacity of EVs to remain in silent circulation in populations.

Human enteroviruses (EVs) belong to the *Enterovirus* genus of the family *Picornaviridae* and considered important causative agents associated with a spectrum of human diseases such as fever, hand-foot-and-mouth disease (HFMD), paralysis, aseptic meningitis, encephalitis, myocarditis and neonatal sepsis.^1^ EVs are small non-enveloped viruses comprising 60 copies each of the capsid proteins VP1, VP2, VP3 and VP4, and enclose a positive-sense, single-stranded RNA genome of 7.4-7.5 kb. A single polyprotein translated from the RNA strand is first cleaved into three polyprotein precursors: P1, P2 and P3. While P1 is processed to yield four structural proteins (VP1-VP4), P2 and P3 are precursors of the nonstructural proteins 2A-2C and 3A-3D, respectively.^2^ Currently, EVs comprise more than 100 types which are classified into 12 species, EV-A to EV-L (https://talk.ictvonline.org/).

Enterovirus C99 (EV-C99) is a newly identified EV serotype within the species *Enterovirus C*. EV-C99 was first isolated in Bangladesh in 2000,^3^ and subsequently reported worldwide, including in non-human primates.^2^ ^,^ ^4^ ^,^ ^5^ ^,^ ^6^ EV-C99 strains have been isolated from both acute flaccid paralysis (AFP) patients and healthy individuals.^5^ ^,^ ^7^ Currently, 15 complete genome sequences of EV-C99 are available in the GenBank database, but no nucleotide sequence has been obtained from Brazil. Echovirus 29 (E29) is a member of species *Enterovirus B*. Since its first report in the 1950s,^8^ E29 has been described globally recovered from both humans and non-human primates, and associated with an array of clinical manifestations ranging from non-specific febrile illnesses to AFP.^9^ ^,^ ^10^ ^,^ ^11^ Although E29 has been described worldwide, there are no reports of its detection in Brazil. Here we report the complete genome sequences of one EV-C99 strain and one E29 strain obtained from children with acute gastroenteritis in the Northern region of Brazil. Phylogenetic analysis was performed for comparison with other previously reported strains.

The BRA/TO-16 (EV-C99) sample was collected in 2013 from a 2-year-old female child in the state of Tocantins and the BRA/PA-29 (E29) sample was collected in 2014 from a 1-year-old male infant in the state of Pará. Both patients were experiencing acute gastroenteritis symptoms, such as diarrhea, vomiting and fever. In addition, no symptoms of classical EV associated syndromes had been observed. The protocol used to perform deep sequencing was a combination of several protocols normally applied to viral metagenomics and/or virus discovery.^12^ In summary, 50 mg of each human fecal sample was diluted in 500 μL of Hanks’ buffered salt solution (HBSS), added to a 2 mL impact-resistant tube containing lysing matrix C (MP Biomedicals, USA), and homogenized in a FastPrep-24 5G Homogenizer (MP biomedicals, USA). The homogenized sample was centrifuged at 12,000×*g* for 10 min, and approximately 300 μL of the supernatant was then percolated through a 0.45 μm filter (Merck Millipore, Billerica, MA, USA) in order to remove eukaryotic and bacterial cell-sized particles. Hundred microliters of cold PEG-it Virus Precipitation Solution (System Biosciences, CA, USA), roughly equivalent to one-fourth of the volume of the tube, was added to the obtained filtrate, and the contents of the tube were gently mixed, then incubated at 4ºC for 24 h. After the incubation period, the mixture was centrifuged at 10,000×*g* for 30 min at 4ºC. Following centrifugation, the supernatant (~350 μL) was discarded. The pellet rich in viral particles was treated with a mixture of nuclease enzymes (14 uni TURBO Dnase and 7 uni RNase Cocktail Enzyme Mix-Thermo Fischer Scientific, CA, USA; 9 uni Baseline-ZERO DNase - Epicentre, WI, USA; 25 Benzonase - Darmstadt, Germany; and 9 RQ1 RNase- Free DNase and 0.09mg RNase A Solution - Promega, WI, USA) in order to digest unprotected nucleic acids. The resulting mixture was subsequently incubated at 37ºC for 2 h.^13^

After incubation, viral nucleic acids were extracted using ZR & ZR-96 Viral DNA/RNA Kit (Zymo Research, CA, USA) according to the manufacturer’s protocol. The cDNA synthesis was performed with AMV Reverse transcription (Promega, WI, USA). A second strand of cDNA was synthetized using DNA Polymerase I Lar e (Klenow) Fragment (Promega, WI, USA). Subsequently, a Nextera XT Sample Preparation Kit (Illumina, CA, USA) was used to construct a DNA library, identified using dual barcodes. For size range, Pippin Prep (Sage Science, Inc.) was used to select a 300 bp insert (range 200-400 bp). The library was deep-sequenced using the HiSeq 2500 Sequencer (Illumina, CA, USA) with 126 bp ends.^13^ Following de novo assembly of short sequence reads longer contigs were made using the customized de novo assembly software described by Deng et al.,^14^ viral sequences were recognized by translating them *in silico* in all six possible reading frames. These virtual protein sequences were then used for similarity searches using BLASTx against the proteins of all viral genomes in GenBank. The bioinformatics pipeline trimed sequences of primers involved in the random *reverse transcription polymerase chain reaction (RT-PCR)*, and kept only a single copy of repeated sequences. Residual sequences from the human genome were also removed to accelerate analyses. Contigs of overlapping short reads were then generated using an in-house hybrid de novo assembler program specially designed for viral metagenomics generating longer contigs to facilitate the recognition of highly divergent viral genomes.^14^ ^,^ ^15^ Then a search was performed for sequence similarity against all annotated viral genomes in GenBank using protein similarity searches (BLASTx). The last, and computationally most demanding step, was to reduce the signal noise by removing from this list of tentative viral hits the sequences with higher levels of similarity to non-viral sequences (based on annotation) in the large NR (non-redundant) GenBank database. This NR database shows the family/genus/species of viruses with similarity to the generated dataset (with adjustable E score ranges). The pipeline was fast and sensitive, allowing even highly divergent viruses with only ~15-20% protein identity (depending on length of contigs) to be recognized.^16^

A total of 1,037,866 and 2,473,062 paired-end reads were obtained from the BRA/TO-16 and BRA/PA-29 samples, respectively. Of the total reads, 4% (n = 41,475) from BRA/TO-16 and 1% (n = 23,224) from BRA/PA-29 showed a BLASTx score (with a coverage of 1.326x and 1.111x, respectively) to EV-C99 or E29, correspondingly. The final genome analysis was performed using Geneious software v9.1.8 (Biomatters Ltd., Auckland, New Zealand). Open reading frames were predicted with the Geneious ORF finder. Based on the bioinformatics pipeline used,^14^ no reads related to human, fungal, or bacterial sequences were obtained.

The publically available typing tool *Enterovirus Genotyping Tool* (https://www.rivm.nl/mpf/typingtool/enterovirus/) was used to assign the genotype/serotype of the study strains, confirming the previous classification into EV-C99 (BRA/TO-16) and E29 (BRA/PA-29) obtained during the BLASTx search. Sequences generated here and a set of cognate sequences of EVs available in GenBank were aligned using the CLUSTAL W algorithm in BioEdit Sequence Alignment Editor Program (version 7.0.5.2). The BRA/TO-16 (EV-C99) strain described in this study was aligned with 15 nearly full-length genome sequences available in the GenBank database. In parallel, the BRA/PA-29 (E29) strain was aligned with the JV-10 strain (AY302552), the only full-length genome sequence available in GenBank, as well as 16 partial sequences, ranging from 2200 to 3543 nucleotide (nt) positions, comprising the entire VP1 protein, region used for EV typing.^17^ Genetic analysis was performed with MEGA software version 6.0.^18^ The Kimura two-parameter substitution model and neighbour-joining method was selected to infer phylogenetic relationships among relevant strains. Nucleotide sequences determined in this study have been deposited in GenBank under the accession numbers MK689070 and MK689071.

The Brazilian EV-C99 sequence we have described (BRA/TO-16) showed 78.6% similarity at nucleotide level (nt) (48.2% aa) compared to the BAN00-10461 prototype strain, and 70.1-83.2% nt similarity (41.2-57% aa) when compared to representative EV-C99 strains detected in China, Republic of Congo, United States, Oman, Bangladesh and Madagascar. The relatively high divergence observed between the Brazilian EV-C99 strain and other EV-C99 isolates available in GenBank could be confirmed in the phylogenetic tree (Fig. A). The nucleotide sequences available for E29 strains differ considerably. The Brazilian E29 (BRA/TO-29) strain we have described displayed 79.3% nt and 45.5% aa identity to the JV-10 prototype strain, and significantly low genetic homology to other E29 strains detected in Asia and Africa (8.1-14.3% nt; 4.3-8.3% aa). Despite the low genetic homology obtained, in the phylogenetic tree, the Brazilian E29 strain clustered with the reported E29 strains with a boostrap value of 85% (Fig. B).

**Figure:**
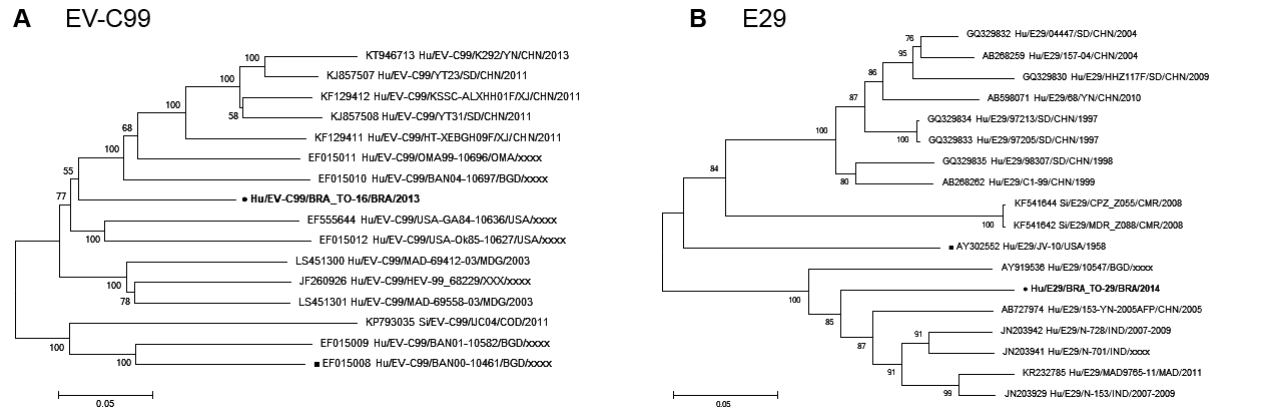
Neighbor-joining phylogenetic tree of nucleotide sequence generate with MEGA 6.0 software of the enterovirus C99 (EV-C99) (A) and echovirus 29 (E29) (B) strains detected from children with acute gastroenteritis in Brazil (highlighted in bold and ●). References of enteroviruses were obtained from GenBank database. Filled black squares label correspond to prototype strains. Accession number, isolates, countries and year of each strain are indicated. The scale indicates the number of divergent nucleotide residues. Bootstrap values are shown at the branch node.

There are various studies describing the epidemiology of EV-C isolates globally,^19^ ^,^ ^20^ including in Brazil;^21^ nevertheless, there are currently very few EV-C99 nucleotide sequences available in the GenBank database. Regardless of the limited number of available strains, they were isolated from samples obtained worldwide, including, Asia, Africa, Europe and North America. This is the first report of EV-C99 in South America, corroborating that EV-C99 has indeed a global distribution, as previously suggested.^2^ The substantial divergence between EV-C99 sequences also suggests the circulation of distinct lineages in different countries;^2^ however, the availability of EV-C99 sequences is still very limited to draw any robust conclusions.

Since the advance of sequencing methods, many newer EV serotypes have been identified, especially those that are “untypeable” by traditional neutralization using standard antisera in cell culture.^2^ In addition, some newly identified EVs, such as EV-C105 and -C116 cannot grow in RD or Hep-2 cells.^22^ Next Generation Sequencing (NGS) is providing a new method for identifying novel EV-C that cannot grow in cell culture.^20^ NGS surveillance described in the present investigation to study enteric viruses, has provided an opportunity to identify the recently described EV-C99 strain for the first time in Brazil. EV-C99 is an emergent EV-C serotype^2^ potentially associated with AFP cases.^7^ ^,^ ^23^ These data, together with the EV-C99 detection reported here, underlines the importance of intensifying non-polio EV monitoring in order to understand the etiological role of EV-C99, as well as other newly discovered EVs, associated with AFP.

Despite E29 being detected globally, epidemiological data on EVs from England, Wales, Spain, Tunisia, USA, India, West Africa and China, reveals that this particular serotype exhibits low rates of prevalence,^9^ ^,^ ^24^ ^,^ ^25^ ^,^ ^26^ ^,^ ^27^ ^,^ ^28^ corroborating its rarity in circulation. The reason for this particular pattern of circulation is still unclear. Both factors associated with the agent and the host probably affect E29 ecology, as observed for many other serotypes.^29^ ^,^ ^30^ Although traditional antisera neutralization methods and molecular approaches are currently available for E29 identification,^8^ ^,^ ^17^ it’s rare pattern of detection seems to impair genetic studies. The poor conclusions drawn from the phylogenetic analyses reﬂect the paucity of data available in the literature regarding E29 sequences. Only one E29 complete genome sequence (JV-10 prototype) is available in the GenBank database, and there are practically no phylogenetic studies looking at this serotype. Here we report the second E29 genome described worldwide.

It is important to mention that the two rare EV strains detected here were isolated from stool samples during a stool sample survey of children suffering from acute gastroenteritis. EVs are classically not known to be associated with acute diarrhea, and the gastroenteritis symptoms observed in the patients BRA/TO-16 and BRA/TO-29 are probably linked to one (or more) of the enteric viruses detected in their fecal samples (i.e., Norovirus and Rotavirus) (data not shown).

Identifying the circulating EVs can help to elucidate the enteroviral biodiversity, improving our understanding of their potential health burden, and enabling a prompt response in case of outbreaks. It is imperative to establish an effective non-polio EV pathogen surveillance system in Brazil. Continuous surveillance of EVs is vital to provide further information on the circulation of EV-C99 and E29 strains, as well as other new or rare EV serotypes in the country. The present study also highlights the capacity of EVs to remain in silent circulation in populations. Surveillance of EV diversity among distinct patient groups, including healthy individuals, together with further surveillance in the surrounding can considerably increase the power of AFP surveillance strategy.

*Ethics* - Previous Ethics Committee approval was granted by Faculdade de Medicina da Universidade de São Paulo (CAAE: 53153916.7.0000.0065), and Centro Universitário Luterano de Palmas ― ULBRA (CAAE: 53153916.7.3007.5516). This was an anonymous unlinked study, and informed consent was not required according to resolution 466/12 concerning research involving humans (Conselho Nacional de Saúde/Ministério da Saúde, Brasília, 2012).
